# Toward an Efficient Differentiation of Two *Diaporthe* Strains Through Mass Spectrometry for Fungal Biotyping

**DOI:** 10.3390/cimb47010053

**Published:** 2025-01-15

**Authors:** Kathleen Hernández-Torres, Daniel Torres-Mendoza, Gesabel Navarro-Velasco, Luis Cubilla-Rios

**Affiliations:** 1 Laboratorio de Bioorgánica Tropical, Facultad de Ciencias Naturales, Exactas y Tecnología, Universidad de Panamá, Panamá 0824, Panama; kathleen-j.hernandez-t@up.ac.pa (K.H.-T.); daniel-t.torres-m@up.ac.pa (D.T.-M.); 2 Programa de Maestría en Microbiología Ambiental, Vicerrectoría de Investigación y Postgrado, Universidad de Panamá, Panamá 0824, Panama; 3 Departamento de Microbiología y Parasitología, Facultad de Ciencias Naturales, Exactas y Tecnología, Universidad de Panamá, Panamá 0824, Panama; 4 Departamento de Química Orgánica, Facultad de Ciencias Naturales, Exactas y Tecnología, Universidad de Panamá, Panamá 0824, Panama; 5 Vicerrectoría de Investigación y Postgrado, Universidad de Panamá, Panamá 0824, Panama; 6 Departamento de Microbiología Humana, Facultad de Medicina, Universidad de Panamá, Panamá 0824, Panama; gesabel.navarro@up.ac.pa; 7 Centro de Investigación e Información de Medicamentos Tóxicos (CIIMET), Facultad de Medicina, Universidad de Panamá, Panamá 0824, Panama; 8 Sistema Nacional de Investigación (SNI), SENACYT, Panama 0816, Panama

**Keywords:** *Diaporthe melongenae*, endophytic fungi, chemotaxonomy, biotyping, culture media, mass spectrometry, MS/MS fragmentation analysis

## Abstract

Considering that fungi display a great morphological, ecological, metabolic, and phylogenetic diversity, their taxonomic identification is extremely important because it helps us establish important information about each species and its possible biochemical and ecological roles. Traditionally, the identification of fungi at the species level has been carried out with molecular tools such as DNA sequencing, but it still represents a huge challenge today due to the heterogeneity of the fungal kingdom, making the task of identification a complex and difficult process. Biotyping, a type of chemotaxonomy, has been developed in the field of the identification/differentiation and classification of micro-fungi through tools such as mass spectrometry (MS). Here, two endophytic strains isolated from two different hosts were cultivated and studied regarding their morphology and molecular biology. Morphology analysis determined the strains as *Diaporthe*, and the molecular analysis results grouped them as *D. melongenae*. We sought a faster and less complex way of differentiating these fungal strains of interest through an MS chemical profile and MS/MS data using a low-resolution mass spectrometer. Additionally, we linked this information with the structure of compounds previously isolated in the genus *Diaporthe*. Studies conducted using this technique allowed us to propose the structure of distinctive molecules that are unique to each strain and share compounds common to this genus (13 compounds in total). In addition, this is the first report of secondary metabolites in *D. melongenae*. The dataset demonstrates that the two strains under investigation can be distinguished via mass spectrometry, suggesting host affinity; both exhibits pronounced differences in their chemical profiles across all culture media and incubation periods with the parameters described herein.

## 1. Introduction

Endophytic fungi are microorganisms that are generally isolated from the healthy tissues of a host plant and can survive inside it without causing apparent symptoms of a disease [1,2,3]. This mechanism of plant–fungus interaction is carried out under specialized chemical signaling processes, establishing cooperation to help maintain the response to abiotic and biotic stress conditions [2,4,5]. Therefore, for both species, these interactions can also include mutualism, parasitism, or commensalism [6,7]. Many of these species have been studied in detail, for instance, in terms of their diversity; defense mechanisms; regulation of virulence factors; and potential in the pharmaceutical, agronomic, and environmental industries. However, most of the research around the world is focused on the biosynthesis of chemical compounds since endophytic fungi have a very versatile metabolism and produce a variety of secondary metabolites [8,9]. Moreover, this approach omits the use of new compounds that have no expected bioactivity and can be discarded.

Considering that fungi display great morphological, ecological, metabolic, and phylogenetic diversity, their taxonomic identification is extremely important because it helps us establish important information about a species and its possible biochemical and ecological roles, such as recognizing beneficial species for humanity or identifying species with harmful functions such as opportunistic pathogens. Traditionally, the identification of fungi at the species level has been carried out using molecular tools such as DNA sequencing [10,11]. However, due to the inherent heterogeneity of the fungal kingdom, this task continues to pose a significant challenge, as exemplified by species within the *Diaporthe* genus, which, in particular, exhibits notable complexity, harboring numerous cryptic species that further complicate the task of an accurate classification [12,13,14,15,16].

Biotyping in the field of identification and classification of micro-fungi has been developed through tools such as mass spectrometry (MS) [17,18]. MS is an analytical technique that detects small molecules with high sensitivity and precision. A notable advantage is that it can be combined with chromatographic techniques, such as liquid chromatography in separation methods (LC-MS), to analyze complex samples such as organic extracts obtained through fungi cultures for measurements based on chemical components, either as markers or contributors to the development of a fingerprint profile [18,19,20,21].

A challenge in characterizing fungal fingerprints is the multiple growth variables that can create unwanted variations, leading to ambiguous identification [17,18,22,23,24,25,26]. The most common parameters in fermentation are the availability of nutrients, pH, temperature, and incubation periods. To carry out biotyping in fungi, the parameters of the cultures must be verified. It is important to define the growth stage at which chemical compounds are produced, not only for maximum production but also to reveal patterns of occurrence. An important challenge for this type of project is the lack of universal sample-handling protocols in mass spectrometry with a sufficient resolution to optimize the identification of fungal species [26,27,28,29].

Members of the genus *Diaporthe* (Family Diaporthaceae, Order Diaporthales and Class Sordariomycetes) have been isolated worldwide and they have been found to colonize a wide range of hosts—mainly as pathogens, nonpathogenic endophytes, or saprobes in plants; and as pathogens in humans and other mammals. According to Species Fungorum (https://www.speciesfungorum.org last accessed 11 December 2024) the genus *Diaporthe* comprises 975 described species, and 886 species have been attributed to the asexual state *Phomopsis*. It has been proven to be a prolific source of bioactive secondary metabolites with potential applications in pharmaceutical and agricultural fields, with polyketides being the largest and most structurally diverse class of secondary metabolites reported. They display biological activities such as antifungal, antibacterial, anti-tuberculosis, antiviral, antioxidant, anti-inflammatory, cytotoxic, and phytotoxic effects, among others [30,31,32,33,34,35]. As a plant pathogen, *Diaporthe* is a genus of great importance, and its classification has been discussed by many researchers based on morphological information, culture characteristics, and host affiliations, but the previous classification is no longer applicable, resulting in a need to reformulate the identification of species in the genus with advanced molecular techniques to solve this identification problem [36]. *D. melongenae* was reported as the pathogen agent in leaf and fruit blight in eggplant cultures in the Philippines and there are no reports of the isolation of secondary metabolites [37,38].

The introduction of rapid and reliable MS-based identification in mycology has progressed at a slower pace due to the biological complexity of filamentous fungi. To address this variability, some studies have focused on standardizing growth conditions and sample preparation to construct chemical profiles and compare them with improved references libraries [39,40]. Due to current trends in the development of potential alternative methods for the rapid description of fungal species [41,42,43,44,45,46], the objective of this study was to define chemical profiles using mass spectrometry data (ESI-TQD-MS/MS) to chemotaxonomically distinguish two *Diaporthe* strains that looked morphologically different but, through microscopy and molecular biology, have been placed in the same clade. The mass of the cationized or anionized molecule ions, obtained for each extract, were linked to the molecular weight and structure of compounds that had been previously isolated or reported in the genus *Diaporthe*.

## 2. Materials and Methods

### 2.1. Selection of Endophytic Fungi and Culture of Strains

Healthy specimens of *Avicennia germinans* (Family: Acanthaceae) and *Rhizophora mangle* (Family: Rhizophoraceae) were collected in the Sarigua National Park, Province of Herrera, Republic of Panama, in 2004 and 2005, respectively. Mature leaves of each plant were surface-sterilized according to our protocol that was previously reported in [47]. From *A. germinans*, we obtained isolate F0728, and from *R. mangle*, the isolate F0891 was successively re-plated until a pure strain was obtained. The pure fungal strains were stored at −80 °C, in a solution of 10% glycerol, and preserved until further use. Both strains were conserved by the International Cooperative Biodiversity Groups program (ICBG-Panama) and the Nagoya Project in Panama. Currently, the strains are kept in the culture collection of the University of Panama.

Before the reactivation of the strains, a culture recovery protocol was applied [48]. Mycelial plugs (5 mm) were aseptically transferred into Petri dishes containing malt extract agar (MEA), incubated at 26 °C from 7 to 15 days, and then stored in sterile distilled water.

### 2.2. Fungal Strains Identification: Morphological and Molecular

The morphological identification was carried out in potato dextrose agar (PDA) cultures. The macroscopic characteristics of the cultures were observed over a 15-day period at 26 °C under permanent illumination. The fungal structures were imaged under a Leica DM200 LED microscope with Plan optics connected to an MC170HD photographic camera (Leica Microsystems, Wetzlar, Germany).

The biological culture for genomic DNA extraction and the molecular identification of each strain were carried out as we previously reported in [49].

Taxonomic identification was assigned according to sequence similarity to public data in NCBI GenBank^®^ and morphological data. The sequence obtained for each strain was deposited in the GenBank^®^ database with the accession numbers PP669695 for strain F0728 and PP669694 for strain F0891. The phylogeny was estimated with the sequences available in public data from NCBI GenBank^®^.

### 2.3. Initial Cultivation and Extraction

The fungal strains were grown under saturated conditions at 26 °C in Petri dishes (100 × 15 mm) in three media—MEA, PDA, and Sabouraud dextrose agar (SDA) (the three media were from BD Difco^TM^, Becton, Dickinson and Company, Sparks, MD, USA)—in an incubation chamber with permanent light. Each medium was prepared according to the manufacturer’s instructions. The fungi were removed at 7, 15, 22, and 30 days of growth; each sample was cut into pieces with a sterile spatula and stored in sterile plastic bags at −20 °C.

The frozen sample was placed in a 1 L beaker with 500 mL of ethyl acetate (ACS grade, Tedia^®^, Tedia Company Inc., Fairfield, OH, USA) for 20 min. Subsequently, the sample was ground using a Polytron^®^ (Brinkmann Instruments, Westbury, NJ, USA) homogenizer. To avoid excessive heat production, this operation was carried out in an ice bath. The homogenized sample was then filtered using filter paper under vacuum. The filtrate was placed in a separatory funnel, and the organic phase was obtained; then, the aqueous phase was extracted with ethyl acetate (2 × 100 mL). The organic phases were combined and placed in a flask to evaporate the solvent under reduced pressure. The extract was re-suspended in methanol and transferred to amber glass vials. Finally, the MeOH was evaporated, and the vial was weighed to determine the mass of the extract. Each extract was obtained in duplicate. In each case, the controls were obtained, and the respective sterile culture media controls were made for the four growth periods.

### 2.4. Analysis of Organic Extracts with LC-MS

The organic extracts were diluted in methanol suitable for LC-MS (LiChrosolv^®^, Merck, EMD Millipore, Billerica, MA, USA) at approximately 1.0 mg/mL for each sample, filtered through 0.22 μm PTFE membrane filters and analyzed in a Waters ACQUITY class-H UPLC^TM^ system using an Acquity UPLC^®^ BEH (1.7 μm) C18, reverse phase LC column, 2.1 × 100 mm, Waters PDA eλ detector, and XEVO^®^ TQD spectrometer (Waters Corporation, Milford, MA, USA) supplied with an electrospray ionization (ESI) source. The gradient for chromatographic separation was carried out using a 10 min step UPLC run of acetonitrile (HPLC/Spectro, Tedia^®^, Tedia Company Inc., Fairfield, OH, USA) and water (H_2_O), starting at 70–30% AcCN-H_2_O and increasing to 100% AcCN in 7.5 min, followed by maintaining 100% AcCN for 1 min before returning to the initial condition of 70–30% AcCN-H_2_O in 1 min until the completion of 10 min, with a flow rate of 0.2 mL/min throughout the run. The ESI-MS parameters were set as follows: capillary voltage, 3.5 kV for the positive mode and 2.5 kV for the negative mode; cone voltage, 20 V; source temperature, 150 °C; desolvation temperature, 450 °C; desolvation gas flow, 600 L/h for the positive mode and 400 L/h for the negative mode; cone gas flow, 50 L/h. Mass spectrometry data were acquired in both modes with a detection range of 125–2000 *m*/*z*. The MassLynx^TM^ software (version 4.2) was used for data acquisition and processing.

### 2.5. ESI-TQD-MS/MS of m/z of Interest

The fragmentation of the *m*/*z* of interest in the ESI-TQD-MS/MS analysis was performed using an XEVO^®^ TQD mass spectrometer (Waters Corporation, Milford, MA, USA). The spectrometer’s parameters were set as follows: capillary voltage, 3.5 kV for positive mode and 2.5 kV for negative mode; cone voltage, 30 V; source temperature, 150 °C; desolvation temperature, 450 °C; desolvation gas flow, 600 L/h; cone gas flow, 50 L/h. The collision energy was set between 15 and 35 V in positive mode and 15 and 25 V in negative mode, depending on the relative abundance of the product ions, which allowed the analysis of the fragmentation mechanism process in the gas phase to have the most accuracy in the identification of compounds. MassLynx^TM^ software (version 4.2) was used for data acquisition and processing.

## 3. Results

### 3.1. Morphological Identification and Molecular Characterization of Fungal Strains

**Figure 1:**
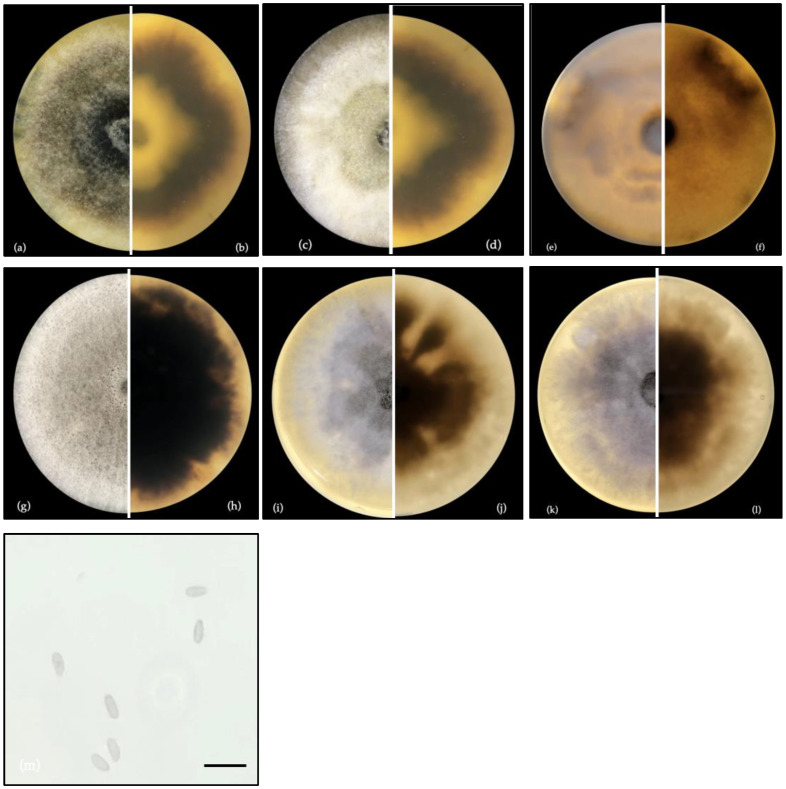
The cultural characteristics of *Diaporthe*. Views of F0728 culture grown on (**a**,**b**) MEA, (**c**,**d**) PDA, (**e**,**f**) SDA from front and reverse; Views of F0891 culture grown on (**g**,**h**) MEA, (**i**,**j**) PDA, (**k**,**l**) SDA from front and reverse. (**m**) Conidia, 10 μm.

Strains were cultured on PDA plates for 15 days at 26 °C, and rapid and abundant mycelial growth was observed. The colonies of F0728 and F0891 were initially flat and had fluffy white mycelium with irregular margins. Both differed from each other in growth over time; F0728 developed rings with slight elevations; the colony turned green and maintained a sterile mycelium for up to 15 days; and, when viewed in reverse, it displayed concentric dark brown to light green margins (Figure 1a–l). Otherwise, the F0891 colony maintained a dense mycelium and turned white gray. The conidia were hyaline (translucent), cylindrical to ellipsoidal in shape, and generally have one or two cells. Their size varies depending on growing conditions, but typically measure between 5 and 10 μm in length (Figure 1m) [37].

**Figure 2:**
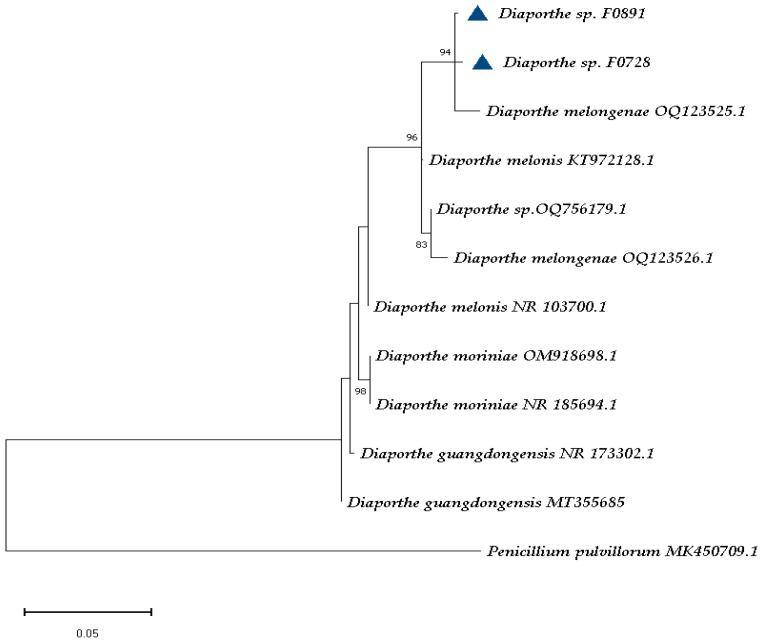
The phylogenetic tree displays the endophytic fungal isolates F0891 and F0791, highlighted in blue, grouped with *Diaporthe melongenae*. The bootstrap support or the node is high (94%), indicating that this grouping is robust and reliable. The ITS region was amplified to facilitate comparisons among species within the *Diaporthe sojae* species complex. A total of eleven strains were included in the analysis. The tree is rooted with *Penicillium pulvillorum* (MK450709.1). The scale bar (0.05) represents the estimated number of nucleotide substitutions per site per branch.

The phylogenetic analysis inferred from the ITS dataset indicated that isolates F0728 and F0891 could be preliminarily classified as *Diaporthe melongenae*, which belongs to or is grouped within the *Diaporthe sojae* species complex. This complex is well known for its agricultural relevance due to its ecological roles as a pathogen, endophyte and saprophyte. For this reason, the phylogenetic tree was constructed using an ITS dataset and ex-type culture sequences to compare strains within the *D. sojae* species complex [37,41]. The inclusion of reference sequences allowed the assessment of whether these endophytic strains are related to previously described species or whether they represent new lineages. The evolutionary relationships were inferred using the Maximum Likelihood method with the Hasegawa–Kishino–Yano model. The tree with the highest log-likelihood (−1856.09) is presented in Figure 2. The percentages of trees on which the associated taxa are clustered together are indicated above the branches. Initial trees for the heuristic search were automatically generated by applying the maximum parsimony method. The tree is scaled, with branch lengths representing the number of substitutions per site. The final dataset included 808 positions, and all evolutionary analyses were performed using MEGA11.

### 3.2. Extraction and UPLC-ESI-MS Profile of Extracts

The fungal strains F0728 and F0891 were cultivated in three different media (MEA, PDA, and SDA) in an incubation chamber at 26 °C for periods of 7, 15, 22, and 30 days, and each was extracted with ethyl acetate to obtain an organic crude extract. The mass data obtained for every crude extract is shown in Table S1.

An aliquot of 1.0 mg of each extract was analyzed with ESI-TQD-MS in both the positive and negative ion mode, and then, the *m*/*z* of interest was fragmented by ESI-TQD-MS/MS to obtain the respective fragmentation spectra. The data obtained for each extract are listed in Table 1 and Table 2.

### 3.3. Identification of Metabolites with ESI-MS/MS

Although the MS data were acquired in low resolution, they could nevertheless be linked to the masses of compounds previously reported in the genus *Diaporthe*. The obtained data were cross-referenced with the Dictionary of Natural Products ver. 32.2 (https://dnp.chemnetbase.com last accessed on 2 February 2024), encompassing all isolated molecules belonging to the genus *Diaporthe*. A literature search allowed us to recognize molecular ions that were present in the extracts of F0728 and F0891, and that matched with the corresponding compounds that were already reported for the genus *Diaporthe*. The ESI-MS experiment registered the presence of [M − H]^−^ in the negative ionization mode and [M + H]^+^ in the positive ionization mode; the relative information of each was employed to determine the molecular weight of the possible compounds.

The cationized or anionized molecule ions that possess a relative abundance equal or superior to 50% and were linked to a previously isolated compound from a species of the genus *Diaporthe* were selected to obtain MS/MS fragmentation information.

The MS/MS experiments and fragmentation pattern analysis facilitated the identification of significant molecular fragments. Their formation from the suggested compound’s structure, following the established fragmentation mechanisms, was well justified. Consequently, this confirmed the presence of compounds that were previously isolated from this genus in a variety of our extracts.

## 4. Discussion

Several growth methods have been reported to determine chemical diversity as a chemotaxonomic tool, but it has not been demonstrated that all culture media and times of incubation can be applied to the characterization of chemical profiles [40,50]. Here, we demonstrated that samples processed using the method described above in triplicate were able to provide optimal conditions for growth, metabolite production, and strain characterization. For fungal identification, morphological techniques such as microcultures were employed, with the incubation parameters being modified to 26 °C for 10 days [51,52].

Our morphological analysis revealed that both strains belonged to the genus *Diaporthe*, with fungal sporulation observed as previously described in [12,13,53]. Morphological indications were further confirmed by the BLAST results and phylogenetic analysis, which showed a 99% sequence match with *D. melongenae* in GenBank^®^.

In the phylogenetic analysis, isolates F0891 and F0791 clearly grouped with *D. melongenae*, but the ITS sequences were not identical, suggesting that the strains may be distinguishable at a molecular level. Therefore, a multigenic phylogenetic analysis, commonly used to improve the resolution and reliability of the observed phylogenetic relationships, is recommended for future taxonomic descriptions [14,16,37,54]. However, biotyping using mass spectrometry emerges as a potentially invaluable tool for accurate characterization. The endophytic isolates used in this study demonstrate the phenomenon of phenotypic plasticity, which allows for significant variability in the production of compounds depending on the environment, host, and other cultivation conditions [55,56,57]. This is reflected in our MS results listed in Table 1 and Table 2, where it can be observed that the variability in the relative abundance and content of ions depends on the culture media and time of incubation. This phenomenon has ecological implications and is also crucial for biotechnological applications, such as the biotyping of filamentous fungi, where manipulating cultivation conditions can optimize the production of bioactive compounds. The technique we employed here offers important advantages by providing detailed chemical compositions of the biochemical components in the growth of both strains, allowing the precise discrimination between strains [18,58].

For both strains studied here, we obtained ESI-MS spectra from different culture extracts, and we analyzed ions corresponding to the molecular mass of compounds that were previously obtained/reported from species belonging to *Diaporthe*. The analysis of the MS and MS/MS data was performed as follows: cationized or anionized molecular ions that were common and detected at least two times in the different extracts with a relative abundance greater than 10% were determined (Table 1 and Table 2). Moreover, cationized or anionized molecular ions with a relative abundance greater than 50% were selected, and if the MS/MS data of parent ions coincided with the molecular ions of compounds that were previously obtained/reported in the genus *Diaporthe*, the ions were analyzed.

First, we focused on a group of ions that were common and appeared at least twice in the different extracts obtained from the various cultures of strains F0728 and F0891, we were able to correlate at least six compounds. For example, ions *m*/*z* 211 and 213 were recorded in ESI (+) (Table 3). Our literature search for genus *Diaporthe* showed that there were just four compounds reported with a molecular mass of 210 (in positive mode, M + H = 211), and there was only one compound reported with a molecular mass of 212 (in positive mode, M + H = 213).

The cultures of both strains on SDA showed the presence of the ion *m*/*z* 211 and a 100% relative intensity in F0891. The MS/MS fragmentation analysis showed that the obtained fragments were exclusively generated from 6-[(1E)-3-hydroxy-1-methyl-1-propenyl]-4-methoxy-3-methyl-2H-pyran-2-one (**1**), which was previously produced by *D. gulyae* and is known as gulypyrone B or nectriapyrone D [59]. We also noted the presence of the ion *m*/*z* 213 for which there is only one structure reported in the literature; the MS/MS fragmentation analysis showed that this ion corresponded to the compound gulypyrone A, 6-[(2S)2-hydroxy-1-methylpropyl]-4-methoxy-5-methyl-2H-pyran-2-one (**2**), which is also produced by *D. gulyae* and was reported as a potential mycoherbicide.

In the case of ions that were common to both strains, as determined via ESI (-), *m*/*z* 193, 213, 235, and 249 were observed. These also corresponded to one or more structures of previously characterized compounds of the genus *Diaporthe*.

For the *m*/*z* 193 ion, there were three structures; however, the formation of a fragment with 31 uma less can only occur in the compound 4-methoxy-3-methyl-6-(1-methyl-1-propenyl)-2*H*-pyran-2-one (**3**), which is known as nectriapyrone, is produced by *D. angelicae* (Anamorph *Phomopsis foeniculi*), and reported as a phytotoxin [60]. The formation of fragments was as follows: *m*/*z* 175 (**3**-H_2_O), *m*/*z* 149 (**3**-CO_2_), *m*/*z* 147 (**3**-OCH_3_ and CH_3_), and *m*/*z* 107 (**3**-OCH_3_ and C_4_H_7_) (Figure S1). This became evident from the corresponding formation mechanisms, justifying their presence in the MS/MS spectrum and largely confirming the presence of this compound in the extracts of both strains.

We found that the molecular ion *m*/*z* 213 may correspond to the compound multiplolide A (**4**), which was previously reported in extracts of *Diaporthe* sp. JC-J7 [61] and *Phomopsis* spp. (strains NXZ-05 [62] and YM 311483 [63]). Meanwhile, the ion *m*/*z* 235 could correspond to the compound Diaporol R (**5**). At least four compounds have been reported for the anionized molecular ion *m*/*z* 249; two of them are stereoisomers [3α-form (diaporol S)/3β-form (3β-hydroxyconfertifolin), see **6**]. Compounds **5** and **6** were reported in the *Diaporthe* sp. associated with *Rhizophora stylosa* [64]. Here, diaporol R showed cytotoxicity against the SW480 cell line. All these six structures ultimately had a terpenoid skeleton.


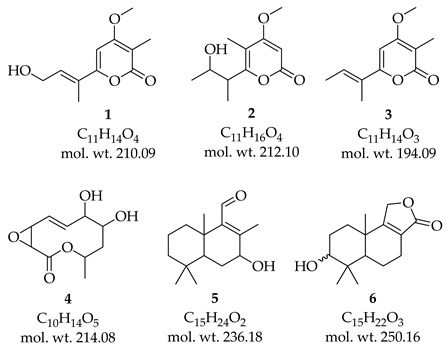


It is evident that all extracts obtained from the different cultures exhibited ions that allowed differentiation between strains. Given the complexity and amount of MS data obtained for each sample, we focused on only cationized or anionized molecular ions with relative intensities greater than 50% (Table S2). The chemical diversity produced in PDA was noteworthy, as evidenced by the quantity of ions produced using ESI in both positive and negative modes. Meanwhile, MEA predominantly yielded ions that were obtained by ESI (-).

We were able to identify another set of seven compounds (**7**–**13**) via MS/MS fragmentation analysis, resulting in a total of thirteen compounds. In Table 4, we show the MS^2^ data obtained for some of the main cationized or anionized molecules from the different cultures; the analysis of the fragmentation mechanism may have justified the formation of parent ion guides for the structures of compounds that were previously isolated/reported from the genus *Diaporthe*. Additionally, Table 4 includes the mass of the fragments, the percentage of relative abundance present in the MS/MS spectra, and the sets of atoms that were detached from the original molecules to form them.

The structures assigned to compounds **7**–**13** were those exhibiting relative abundances equal to or exceeding 50% in their mass spectra. Furthermore, the formation of fragments that were observed or derived through MS/MS could subsequently be correlated with a chemical framework whose genesis from the respective compound’s structure was explicable via fragmentation mechanisms. For the molecular ion *m*/*z* 189 [M + H]^+^, there was one compound, 4-ethyltetrahydro-3-methyl-5-propyl-2,3-furandiol (**7**), reported from *Diaporthe* sp. XZ-07. We found that molecular ion *m*/*z* 195 [M − H]^−^ was consistent with a compound also reported in *Diaporthe* sp. XZ-07, named dihydro-5-[5-(1-hydroxyethyl)furan-2-yl]-2(3H)-furanone (**8**) [65].


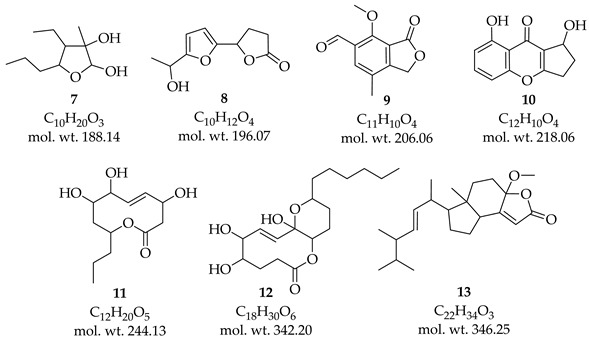


The chemical structure of the molecular ion *m*/*z* 207 [M + H]^+^ was related to diaporthelactone (**9**), which was reported in *Diaporthe* sp. HLY2 and was obtained as an endophyte from the leaves of *Kandelia candel*, showing moderate cytotoxicity against KB and Raji cell lines [66]. The search for molecular ion *m*/*z* 217 [M − H]^−^ resulted in just one hit and matched with the compound diaportheone A (**10**), obtained from *Diaporthe* sp. P133, an endophytic fungus from the leaves of *Pandanus amaryllifolius*, displaying antitubercular activity against *M. tuberculosis* H_37_Rv [67]. We found just one hit for the molecular ion *m*/*z* 245 [M + H]^+^, and this resulted in the structure of phomolide G (**11**), which was previously reported for *D. terebinthifolli* GG3F6 and *Phomopsis* sp. A123 [68]. For the molecular ion *m*/*z* 343 [M + H]^+^, we found one result; the fragmentation analysis was consistent for the structure of phomolide C (**12**) which was reported for *Diaporthe* sp. 1308-05 and *Phomopsis* sp. B27 [69,70]. For the molecular ion *m*/*z* 345 [M − H]^−^, there was one result, and it corresponded to the compound volemolide (**13**), which was reported in *Diaporthe* sp. LG23 from the leaves of *Mahonia fortunei* [71]. All these seven structures ultimately had a polyketide skeleton.

Thus, these results represent the first report of secondary metabolites in strains of *D. melongenae* which were obtained as endophytes from two different mangrove hosts.

**Figure 3:**
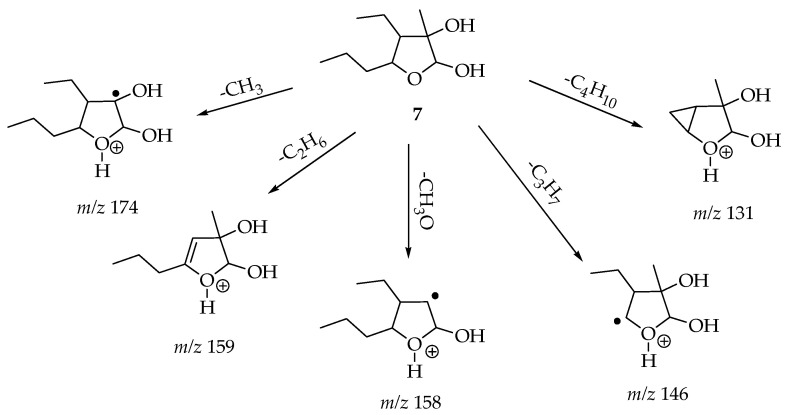
Proposed structures for each product ion of the cationized molecules of compound **7**, *m*/*z* 189 [M + H]+.

**Figure 4:**
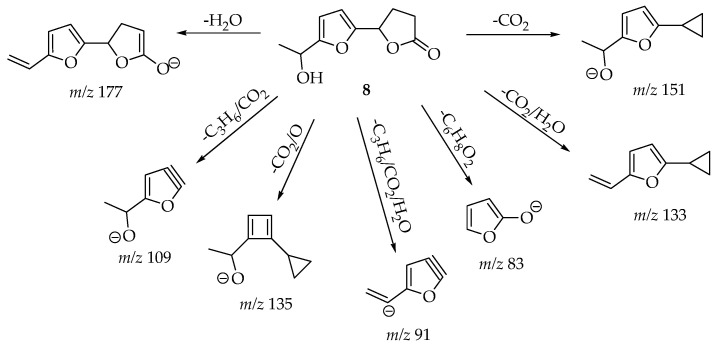
Proposed structures for each product ion of the anionized molecules of compound 8, *m*/*z* 195 [M − H]−.

**Figure 5:**
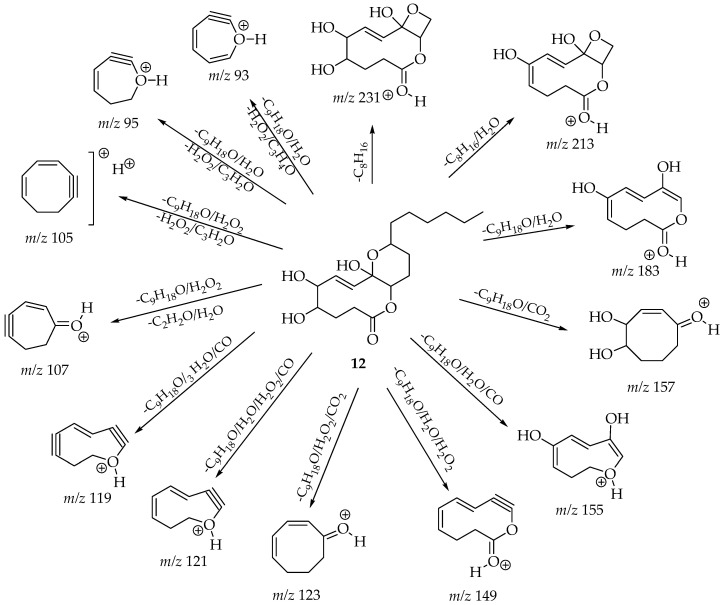
Proposed structures for each product ion of the cationized molecules of compound 12, *m*/*z* 343 [M + H]+.

Figure 3, Figure 4 and Figure 5 exemplify the genesis of principal fragments that were evident in the MS/MS spectra for compounds **7**, **8**, and **12** (Figures S2–S4); they are presented merely as illustrative instances, as this manuscript does not endeavor to elucidate the genesis of all fragments across all compounds. Each product ion corresponds to the sequential or concerted loss of molecular fragments, evidencing higher intensities when the anion or cation is localized on the most stable position.

## 5. Conclusions

To enhance the reliability and accuracy of this study, it is crucial to integrate multiple identification techniques. Combining morphological, molecular, and MS data approaches provides a comprehensive understanding of fungal strains and facilitates a more robust characterization. In order to characterize our strains, we applied various methods, including morphological, molecular biology, and mass spectrometry data, to chemotaxonomy. Among these methods, MS and the morphological analysis proved valuable in differentiating between F0728 and F0891, revealing pronounced differences in their chemical profiles across all culture media and incubation periods with the parameters described herein. Also, there are shared compounds between both strains, as well as unique ones, along with compounds that are common to other species within the *Diaporthe* genus. The MS data also suggests the likely existence of some compounds that have not been previously described. In contrast, the molecular analysis showed strain similarity within the *Diaporthe melongenae* cluster.

The phenomenon of phenotypic plasticity has ecological implications, and it is also crucial for biotechnological applications such as biotyping of filamentous fungi. Thus, by comparing the mass or chemical profiles, unique patterns can be identified that reflect the specific molecular composition of each fungal species. The technique we employ offers significant advantages by providing the detailed chemical compositions of the biochemical components in the growth of both strains, allowing for the accurate discrimination between them [18,58].

By taking advantage of the strengths of different methodologies, we were able to address the limitations of each technique, improving the resolution for strain differentiation. This integrative strategy ensures a deeper understanding of the ecological roles of fungal strain and the biochemical potential under study, ultimately contributing to a more comprehensive view on this topic.

This approach is still under development but holds great potential as a complement to other molecular identification techniques such as PCR, qPCR, or LAMP. Therefore, our approach has practical merits and experimental conditions that allow the identification and/or differentiation between strains. As more databases and standardized methodologies are developed, the resolution and accuracy of the method could be improved, successfully integrating this strategy into fungal identification workflows [18,39].

## Figures and Tables

**Table 1:** The *m*/*z* values of the cationized molecules determined via ESI (+) and their relative abundances *.

Culture Media	Period of Incubation	F0728	F0891
MEA	7 days	163 (100) **; 177 (56.8); 211 (28.4); 213 (34.6); 215 (58.8); 307 (13.2); 325 (14.3)	159 (46.6); 180 (87.6); 183 (100); 193 (80.3); 207 (32.1); 211 (69.4); 229 (19.0); 245 (17.9); 251 (14.8) 268 (14.8); 279 (13.0); 331 (10.2); 340 (11.2); 384 (11.2)
	15 days	163 (23.3); 177 (100); 191 (52.3); 207 (13.6); 213 (10.8)	159 (37.9); 185 (100); 211 (25.3); 213 (26.7); 279 (15.0) 307 (11.4); 321 (68.5); 343 (65.4); 351 (16.3); 365 (12.3)
	22 days	163 (16.5); 177 (100); 191 (60.6); 205 (15.8); 207 (16.1)	159 (23.4); 185 (100); 207 (58.0); 213 (33.1); 243 (23.7); 251 (20.0); 321 (44.0); 343 (32.9)
	30 days	175 (48.1); 177 (35.7); 179 (68.6); 187 (100); 196 (39.8); 213 (25.5); 233 (39.3); 277 (24.5); 317 (39.0); 333 (19.0); 390 (10.5)	159 (17.6); 180 (22.7); 185 (100); 207 (40.5); 213 (28.3); 243 (19.8); 321 (43.4); 343 (43.1); 365 (12.8); 379 (12.7)
PDA	7 days	160 (100); 163 (52.0); 171 (51.5); 177 (50.1); 180 (37.9); 189 (35.2); 215 (46.8); 227 (14.6); 345 (10.4)	180 (27.1); 195 (89.8); 213 (58.7); 295 (18.0); 331 (12.5); 348 (19.0); 351 (18.0; 353 (100)
	15 days	160 (79.3); 163 (94.5); 171 (44.36); 177 (100); 189 (72.02); 191 (40.1); 213 (47.5); 215 (58.1); 259 (19.9); 309 (28.1); 325 (18.6)	180 (50.4); 195 (53.3); 211 (40.2); 213 (34.3); 268 (24.2); 295 (12.0) 351 (21.0); 353 (100)
	22 days	160 (52.4); 163 (100); 177 (62.7); 189 (30.3); 191 (19.4); 211 (14.2); 213 (21.2); 215 (20.3); 309 (16.5)	165 (23.5); 180 (27.1) 185 (34.1); 193 (58.0); 195 (92.2); 213 (51.9); 233 (23.9); 268 (40.5); 351 (28.7); 353 (100)
	30 days	163 (100); 177 (57.5); 191 (89.5); 207 (76.3); 213 (96.6); 215 (64.0); 229 (45.7); 259 (58.0); 275 (29.7); 307 (20.7); 359 (23.5); 413 (17.3); 429 (10.2)	165 (33.8); 183 (21.8); 193 (62.2); 195 (57.0); 211 (16.6); 213 (28.8); 237 (21.4); 268 (21.2); 351 (24.2); 353 (100)
SDA	7 days	160 (100); 163 (39.7); 171 (41.4); 189 (41.5); 211 (24.4); 215 (64.0) 265 (25.1); 309 (16.5); 325 (10.9); 337 (12.5); 353 (11.3)	180 (23.5); 197 (47.8); 211 (100); 245 (58.3); 261 (13.3)
	15 days	160 (100); 163 (21.7); 164 (29.8); 175 (36.0); 189 (48.9); 211 (23.6); 215 (26.5)	164 (27.4); 180 (50.0; 188 (73.4); 197 (45.4); 211 (100); 245 (57.6); 261 (19.7); 353 (31.8)
	22 days	160 (100); 163 (51.0); 171 (37.2); 175 (36.0); 183 (34.3); 189 (52.1); 193 (41.8); 211 (30.4); 213 (34.0); 215 (80.7); 228 (29.0); 265 (20.0); 309 (25.0)	164 (47.0); 180 (45.0); 197 (34.7); 211 (100); 245 (64.8); 261 (18.7); 353 (33.2)
	30 days	160 (100); 163 (51.7); 171 (32.4); 177 (35.3); 189 (52.8); 211 (27.3); 213 (31.0); 215 (70.0); 265 (26.5); 309 (22.7)	164 (57.0); 180 (44.8); 185 (30.6); 197 (38.5); 211 (100); 245 (61.8); 353 (33.8)

* For *m*/*z* values above 10% of the relative abundance; ** relative abundance in %.

**Table 2:** The *m*/*z* values of the anionized molecules determined via ESI (-) and their relative abundances *.

Culture Media	Period of Incubation	F0728	F0891
MEA	7 days	169 (55.7) **; 179 (57.6); 193 (58.3); 195 (100); 209 (66.1); 213 (20.9); 235 (15.7); 279 (16.8); 349 (20.1); 357 (18.0); 371 (11.5)	155 (100); 173 (20.0); 199 (19.7); 217 (12.5); 249 (82.5); 329 (27.2); 385 (11.5); 505 (16.7)
	15 days	169 (20.9); 193 (17.2); 209 (30.1); 211 (27.7); 213 (16.7); 345 (19.8); 363 (100); 365 (50.1); 378 (34.9); 394 (38.0) 396 (50.1)	155 (100); 173 (92.7); 185 (34.9); 199 (49.8); 213 (30.2); 227 (20.9); 249 (29.2); 319 (21.2); 329 (26.7); 337 (15.3); 505 (12.9); 709 (23.1)
	22 days	167 (42.5); 169 (59.6); 193 (44.7); 209 (59.7); 211 (67.9); 217 (100); 249 (31.2); 345 (66.2); 363 (59.6); 365 (24.5); 379 (20.2); 396 (23.7)	155 (70.0); 173 (100); 185 (38.9); 199 (59.6); 213 (30.0); 227 (25.8); 241 (27.0); 249 (24.4); 269 (22.1); 279 (19.9); 319 (27.5); 329 (53.3); 489 (13.2); 505 (17.1)
	30 days	165 (20.3); 206 (16.9); 251 (25.7); 291 (74.1); 363 (100); 379 (25.1); 415 (74.9); 469 (14.3); 499 (12.2); 612 (25.0)	155 (95.1); 173 (100); 185 (43.4); 199 (72.0); 213 (57.0); 225 (35.0); 241 (48.8); 249 (36.0); 255 (33.9); 269 (32.8); 283 (26.4); 319 (79.0); 329 (63.4); 337 (42.9); 353 (26.6); 473 (26.0); 489 (17.6); 505 (40.8); 533 (27.6); 537 (17.4); 547 (14.4); 577 (14.0)
PDA	7 days	162 (18.4); 169 (13.0); 193 (34.0); 195 (30.3); 209 (27.0); 285 (14.3); 363 (100); 365 (18.2)	327 (16.2); 329 (100); 330 (13.1)
	15 days	169 (29.6); 179 (100); 193 (38.8); 195 (75.3); 197 (62.4); 209 (34.5); 217 (19.7); 249 (18.9); 347 (14.2); 357 (14.3)	235 (25.6); 249 (100); 251 (49.2); 327 (16.6); 329 (98.9)
	22 days	169 (82.0); 195 (100); 209 (68.3); 213 (25.9); 248 (23.5); 331 (21.8); 347 (56.2); 385 (17.8); 407 (38.2); 417 (19.0)	235 (32.7); 249 (100); 251 (74.2); 327 (13.6); 329 (78.5)
	30 days	179 (31.0); 193 (49.6); 197 (95.5); 209 (24.5); 217 (100); 235 (20.0) 249 (32.3); 337 (11.0)	235 (69.0); 249 (100); 251 (21.1); 267 (20.9); 329 (72.2)
SDA	7 days	165 (25.4); 193 (13.6); 204 (15.3); 209 (100); 211 (14.6)	165 (100); 193 (10.6); 251 (11.1); 346 (21.5)
	15 days	165 (12.9); 193 (15.6); 204 (11.0); 209 (100); 211 (45.1)	155 (11.5); 178 (10.6); 259 (18.2); 275 (10.0); 329 (83.3); 346 (35.5); 390 (10.0)
	22 days	165 (27.5); 193 (27.0); 204 (100)	173 (10.0); 259 (10.0); 281 (10.0); 329 (42.9); 346 (10.0); 390 (10.0)
	30 days	165 (26.3); 193 (19.2); 204 (100); 209 (92.6); 211 (28.4)	173 (10.0); 259 (10.0); 329 (46.8); 346 (10.0)

* For *m*/*z* values above 10% of the relative abundance; ** relative abundance in %.

**Table 3:** Common ions obtained among the two studied *Diaporthe melongenae* strains and their relative abundances in parentheses.

Culture Media	Time of Culture	F0728		F0891	
		ESI (+)	ESI (-)	ESI (+)	ESI (-)
	7 days	211 (28.4)	--------	211 (69.4)	--------
MEA	15 days	213 (10.8)	213 (16.7)	213 (26.7)	213 (30.2)
	22 days	--------	249 (31.2)	--------	249 (24.4)
	30 days	213 (25.5)	--------	213 (28.3)	--------
	7 days	180 (37.9)	--------	180 (27.1)	--------
PDA	15 days	213 (47.5)	249 (18.9)	213 (34.3)	249 (100)
	22 days	213 (21.2)	--------	213 (51.9)	--------
	30 days	213 (96.6)	235 (20.0); 249 (32.3)	213 (28.8)	235 (69.0); 249 (100)
	7 days	211 (24.4)	193 (13.6)	211 (100)	193 (10.6)
SDA	15 days	211 (23.6)	--------	211 (100)	--------
	22 days	211 (30.4)	--------	211 (100)	--------
	30 days	211 (27.3)	--------	211 (100)	--------

**Table 4:** MS^2^ data obtained for some of the main cationized or anionized molecules.

Compound	Precursor Ion	Product Ions (*m*/*z*) and Lost Fragments
**1** [ESI (+)]	**211** [M + H]^+^	**196** (4) * [-CH_3_]; **183** (5) [-CO]; **141** (35) [-C_4_H_6_O]; **123** (70) [-C_4_H_6_O/H_2_O]; **97** (20) [-C_4_H_6_O/CO_2_]; **91** (52) [-C_4_H_6_O/H_2_O/OHCH_3_]; **70** (73); **57** (92)
**2** [ESI (+)]	**213** [M + H]^+^	**195** (30) [-H_2_O]; **185** (2) [-CO]; **181** (5) [-HOCH_3_]; **177** (5) [-2H_2_O]; **167** (12) [-C_2_H_6_O]; **153** (55) [-CH_3_OH/CO]; **149** (35) [-C_2_H_6_O/H_2_O]; **109** (38) [-C_4_H_9_O/OCH_3_]; **107** (60) [-C_2_H_4_O/CO_2_/H_2_O]; **95** (100) [-C_4_H_8_O/C_2_H_6_O]; **69** (73) [-C_4_H_8_O/C_2_H_6_O/C_2_H_2_]; **57** (92)
**3** [ESI (-)]	**193** [M − H]^−^	**175** (55) [-H_2_O]; **149** (38) [-CO_2_]: **147** (52) [-OCH_3_/CH_3_]; **131** (30) [-H_2_O/CO_2_]; **107** (23) [-C_4_H_7_/OCH_3_]
**4** [ESI (+)]	**215** [M + H]^+^	**171** (4) [-CO_2_]; **153** (5) [-CO_2_/H_2_O]; **151** (22) [-CO/2H_2_O]; **139** (100) [-C_2_H_2_O/H_2_O_2_]; **97** (18) [-C_3_H_6_/C_2_H_2_O/H_2_O_2_]
**5** [ESI (-)]	**235** [M − H]^−^	**193** (8) [-C_3_H_6_]; **191** (100) [-CHO/CH_3_]; **190** (31) [-C_3_H_9_]; **149** (5) [-C_3_H_6/_CHO/CH_3_]; **146** (2) [-C_4_H_12_/CHO]; **123** (8) [-C_6_H_8_O_2_]
**6** [ESI (-)]	**249** [M − H]^−^	**205** (100) [-CO_2_]; **203** (30) [-CO/H_2_O]; **189** (12) [-CO_2_/H_2_O]; **189** (12) [-CO_2_/H_2_O]; **187** (6) [-CO/2H_2_O]; 163 (8); 135 (12)
**7** [ESI (+)]	**189** [M + H]^+^	**174** (100) [-CH_3_]; **159** (38) [-C_2_H_6_]; **158** (5) [-CH_3_O]; **146** (5) [-C_3_H_7_]; **131** (12) [-C_4_H_10_]
**8** [ESI (-)]	**195** [M − H]^−^	**177** (6) [-H_2_O]; **151** (100) [-CO_2_]; **135** (32) [-CO_2_/O]; **133** (6) [-CO_2_/H_2_O]; **109** (30) [-C_3_H_6_/CO_2_]; **91** (8) [-C_3_H_6_/CO_2_/H_2_O]; **83** (8) [-C_5_H_8_/CO_2_]
**9** [ESI (+)]	**207** [M + H]^+^	**192** (100) [-CH_3_]; **191** (4) [-O]; 174 (4) [-CH_3_]; **164** (6) [-CH_3_/CO]; **163** (5) [-CO_2_]
**10** [ESI (-)]	**217** [M − H]^−^	**173** (68) [-C_2_H_4_O/H^+^]; **158** (100) [-C_2_H_3_O_2_/H^+^]
**11** [ESI (+)]	**245** [M + H]^+^	**159** (18) [-C_5_H_10_O]; **153** (16) [-C_2_H_4_/CO/2H_2_O]; **151** (62) [-C_2_H_6_/CO/2H_2_O]; **139** (100) [-C_3_H_8_/CO_2_/H_2_O]
**12** [ESI (+)]	**343** [M + H]^+^	**231** (18) [-C_8_H_16_]; **213** (14) [-C_8_H_16_/H_2_O]; 183 (14) [-C_9_H_18_O/H_2_O]; **157** (25) [-C_9_H_18_O/CO_2_]; **155** (25) [-C_9_H_18_O/H_2_O/CO]; **149** (48) [-C_9_H_18_O/H_2_O/H_2_O_2_]; **123** (42) [-C_9_H_18_O/H_2_O_2_/CO_2_]; **121** (60) [-C_9_H_18_O/H_2_O/H_2_O_2_/CO]; **119** (84) [-C_9_H_18_O/3H_2_O/CO]; **107** (72) [-C_9_H_18_O/H_2_O_2_/C_2_H_2_O/H_2_O]; **105** (76) [-C_9_H_18_O/H_2_O_2_/CO_2_/H_2_O]; **95** (78) [-C_9_H_18_O/H_2_O/H_2_O_2_/C_3_H_2_O]; **93** (100) [-C_9_H_18_O/H_2_O/H_2_O_2_/C_3_H_4_O]
**13** [ESI (-)]	**345** [M − H]^−^	**327** (6) [-H_2_O]; 301 (13) [-CO_2_]; **285** (16) [-CO/CH_3_OH]; **276** (36) [-C_5_H_9_]; **260** (16) [-C_5_H_10_/CH_3_]; **259** (60) [-C_5_H_11_/CH_3_]; 257 (27) [-C_3_H_8_/CO_2_]; **215** (100) [-C_5_H_11_/CH_3_/CO_2_]; 214 (62) [-C_5_H_12_/CH_3_/CO_2_]

* Relative abundance in %.

## Data Availability

The DNA sequences are deposited in GenBank^®^ (https://ncbi.nlm.nih.gov/genbank (accessed on 5 December 2024)). The data presented in this study are available in the main article and the Supplementary Materials.

## Supplementary Materials

The following supporting information can be downloaded at: https://www.mdpi.com/article/10.3390/cimb47010053/s1.
