# Supplementary material for: Toward an Efficient Differentiation of Two Diaporthe Strains Through Mass Spectrometry for Fungal Biotyping
Source: Curr Issues Mol Biol. 2025 Jan 15;47(1):53. doi: 10.3390/cimb47010053 (PMC11763496; doi:10.3390/cimb47010053)

current issues in molecular biology

MDPI

*Supplementary Information*

# Toward an Efficient Differentiation of Two *Diaporthe* strains through Mass Spectrometry for Fungal Biotyping

**Kathleen Hernández-Torres [1,2,3], Daniel Torres-Mendoza [1,4,5], Gesabel Navarro-Velasco [6,7] and Luis Cubilla-Rios [1,*]**

[1] Laboratorio de Bioorgánica Tropical, Facultad de Ciencias Naturales, Exactas y Tecnología, Universidad de Panamá, Panamá 0824, Panamá; kathleen-j.hernandez-t@up.ac.pa (K.H.-T.), daniel-t.torres-m@up.ac.pa (D.T.-M.)
[2] Programa de Maestría en Microbiología Ambiental, Vicerrectoría de Investigación y Postgrado, Universidad de Panamá, Panamá 0824, Panamá.
[3] Departamento de Microbiología y Parasitología, Facultad de Ciencias Naturales, Exactas y Tecnología, Universidad de Panamá, Panamá 0824, Panamá.
[4] Departamento de Química Orgánica, Facultad de Ciencias Naturales, Exactas y Tecnología, Universidad de Panamá, Panamá 0824, Panamá.
[5] Vicerrectoría de Investigación y Postgrado, Universidad de Panamá, Panamá 0824, República de Panamá.
[6] Departamento de Microbiología Humana, Facultad de Medicina, Universidad de Panamá, Panamá 0824, Panamá; gesabel.navarro@up.ac.pa (G.N.-V.)
[7] Centro de Investigación e Información de Medicamentos Tóxicos (CIIMET), Facultad de Medicina, Universidad de Panamá, Panamá 0824, Panamá.
* Correspondence: luis.cubilla@up.ac.pa; Tel.: +507-6676-5824

**Table S1.** Total amount of organic extracts produced by strains F0728 and F0891

| | Culture Media | Time of culture | Extract 1 in grams | Extract 2 in grams | Average mass in grams |
|---|---|---|---|---|---|
| **F0728** | MEA | 07 days | 15.8 | 10.0 | 12.90 |
| | | 15 days | 8.3 | 4.4 | 6.35 |
| | | 22 days | 9.2 | 7.5 | 8.35 |
| | | 30 days | 6.3 | 1.1 | 3.70 |
| | PDA | 07 days | 5.9 | 5.3 | 5.60 |
| | | 15 days | 4.6 | 9.9 | 7.25 |
| | | 22 days | 13.1 | 7.7 | 10.4 |
| | | 30 days | 7.3 | 5.3 | 6.30 |
| | SDA | 07 days | 6.6 | 5.1 | 5.85 |
| | | 15 days | 5.5 | 7.1 | 6.30 |
| | | 22 days | 8.1 | 8.1 | 8.10 |
| | | 30 days | 2.7 | 2.8 | 2.75 |
| **F0891** | MEA | 07 days | 4.5 | 3.8 | 4.15 |
| | | 15 days | 2.7 | 1.8 | 2.25 |
| | | 22 days | 6.5 | 5.3 | 5.90 |
| | | 30 days | 1.5 | 1.3 | 1.40 |
| | PDA | 07 days | 5.8 | 5.5 | 5.65 |
| | | 15 days | 4.5 | 4.2 | 4.35 |
| | | 22 days | 12.1 | 13.4 | 12.75 |
| | | 30 days | 1.2 | 6.3 | 3.75 |
| | SDA | 07 days | 7.3 | 9.6 | 8.45 |
| | | 15 days | 6.7 | 6.3 | 6.50 |
| | | 22 days | 3.7 | 4.7 | 4.20 |
| | | 30 days | 1.5 | 1.2 | 1.35 |

**Table S2.** List of *m/z* values for the two *Diaporthe melongenae* with a relative abundance above 50%.

| Culture Media | Time of culture | F0728 | | F0891 | |
|---|---|---|---|---|---|
| | | ESI (+) | ESI (-) | ESI (+) | ESI (-) |
| MEA | 07 days | 163 (100)*; 177 (56.8); 215 (58.8), | 169 (55.7); 179 (57.6); 193 (58.3); 195 (100); 209 (66.1) | 180 (87.6); 183 (100); 193 (80.3); 211 (69.4) | 155 (100); 249 (82.5) |
| | 15 days | 177 (100); 191 (52.3) | 363 (100); 365 (50.1); 396 (50.1) | 185 (100); 321 (68.5); 343 (65.4) | 155 (100); 173 (92.7) |
| | 22 days | 177 (100); 191 (60.6) | 169 (59.6); 209 (59.7); 211 (67.9); 217 (100); 345 (66.2); 363 (59.6) | 185 (100); 207 (58.0) | 155 (70.0); 173 (100); 199 (59.6); 329 (53.3) |
| | 30 days | -------- | 291 (74.1); 363 (100); 415 (74.9) | 185 (100) | 155 (95.1); 173 (100); 199 (72.0); 213 (57.0); 319 (79.0); 329 (63.4) |
| PDA | 07 days | 160 (100); 163 (52.0); 171 (51.5); 177 (50.1) | 363 (100) | 195 (89.8); 213 (58.7); 353 (100) | 329 (100) |
| | 15 days | 160 (79.3); 163 (94.5); 177 (100); 189 (72.0); 215 (58.1) | 179 (100); 195 (75.3); 197 (62.4) | 180 (50.4); 195 (53.3); 353 (100) | 249 (100); 329 (98.9) |
| | 22 days | 160 (52.4); 163 (100); 177 (62.7) | 169 (82.0); 195 (100); 209 (68.3); 347 (56.2) | 193 (58.0); 195 (92.2); 213 (51.9); 353 (100) | 249 (100); 251 (74.2); 329 (78.5) |
| | 30 days | 163 (100); 177 (57.5); 191 (89.5); 207 (76.3); 213 (96.6); 215 (64.0); 259 (58.0) | 197 (95.5); 217 (100) | 193 (62.2); 195 (57.0); 353 (100) | 235 (69.0); 249 (100); 329 (72.2) |
| SDA | 07 days | 160 (100); 215 (64.0) | 209 (100) | 211 (100); 245 (58.3) | 165 (100) |
| | 15 days | 160 (100) | 209 (100) | 180 (50.0); 188; (73.4); 211 (100); 254 (57.6 | 329 (83.3) |
| | 22 days | 160 (100); 163 (51.0); 189 (52.1); 215 (80.7) | 204 (100); 209 (84.7) | 211 (100); 245 (64.8) | -------- |
| | 30 days | 160 (100); 163 (51.7); 189 (52.8; 215 (70.0) | 204 (100); 209 (92.6) | 164 (57.0); 211 (100); 245 (61.8) | -------- |

*Relative abundance in %

**Figure S1.** ESI-MS/MS spectra of *m/z* 193 $[M - H]^-$. Collision energy at 20 V.

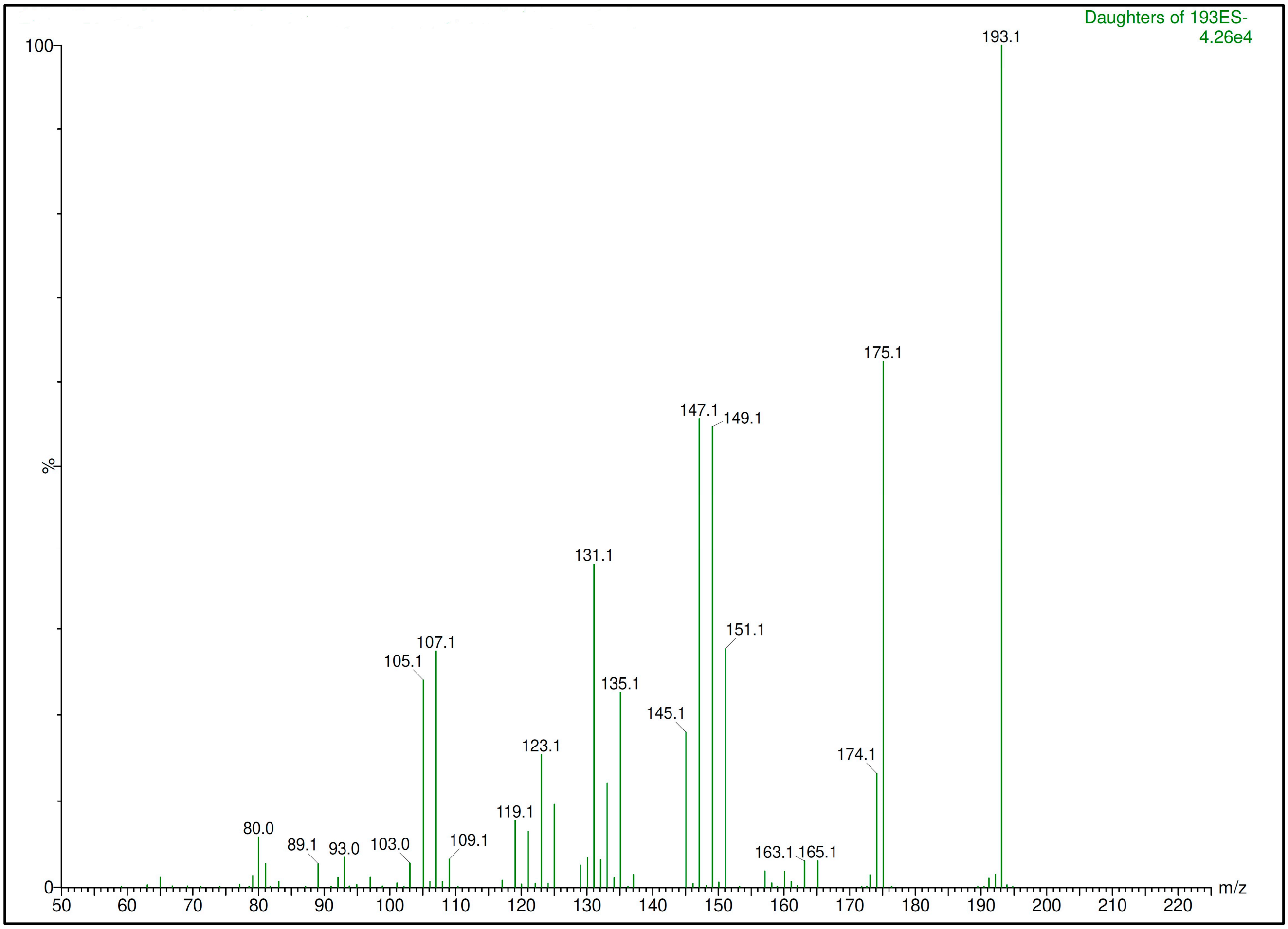

**Figure S2.** ESI-MS/MS spectra of *m/z* 189 $[M + H]^+$. Collision energy at 20 V

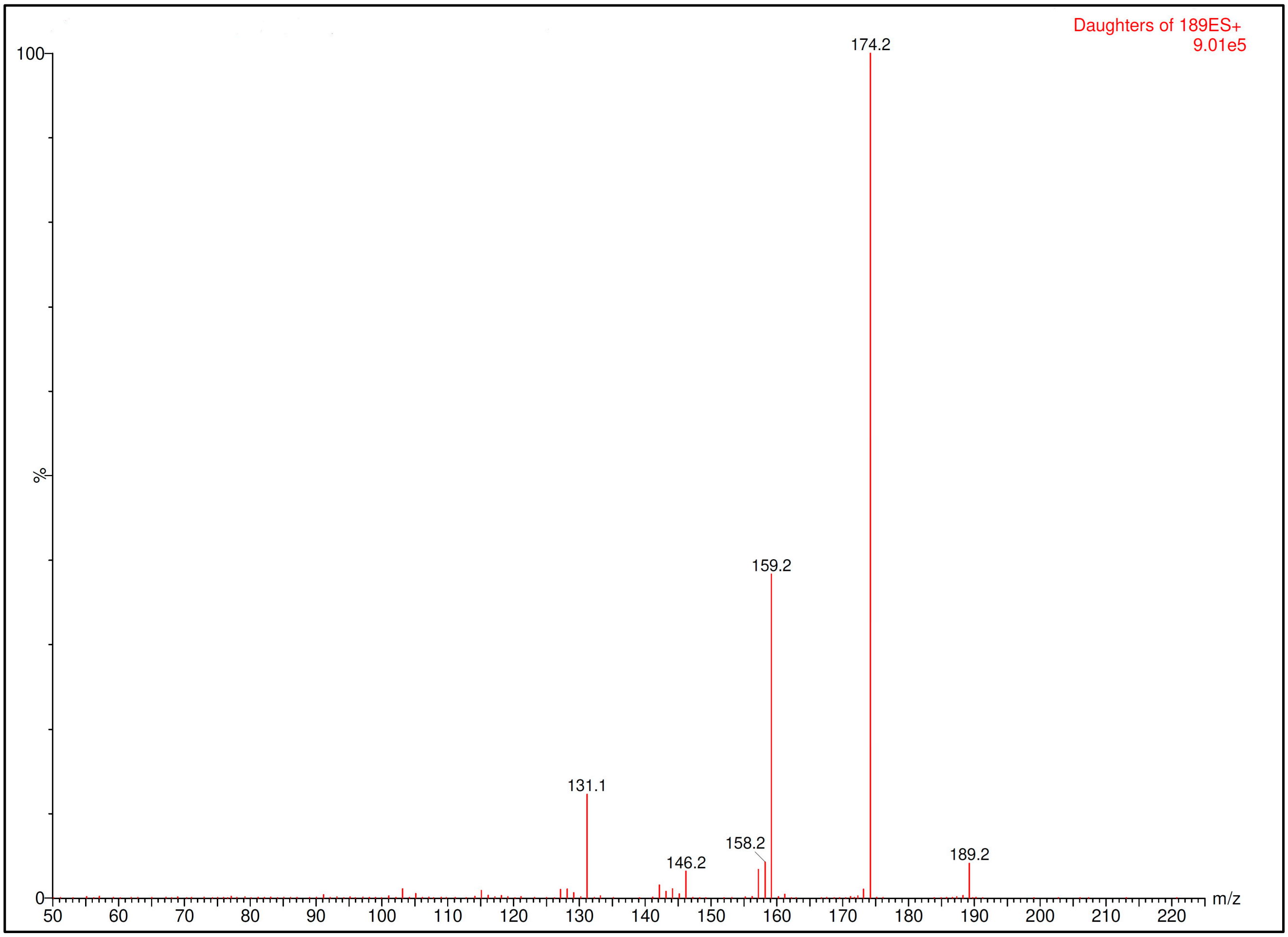

**Figure S3.** ESI-MS/MS spectra of *m*/*z* 195 [M – H]⁻. **A.** Collision energy at 15 V. **B.** Collision energy at 20 V.

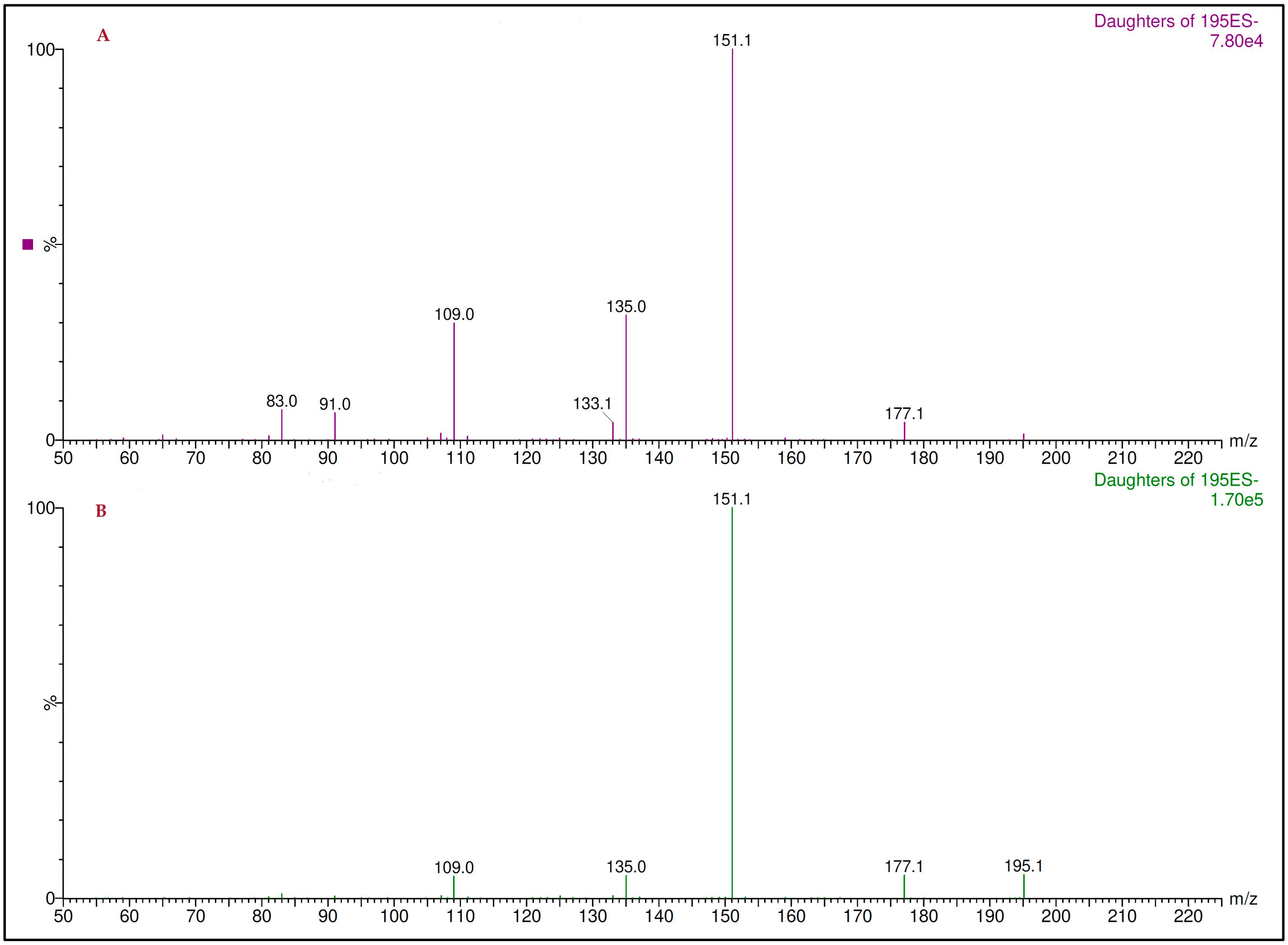

**Figure S4.** ESI-MS/MS spectra of m/z 343 [M + H]+. Collision energy at 32 V

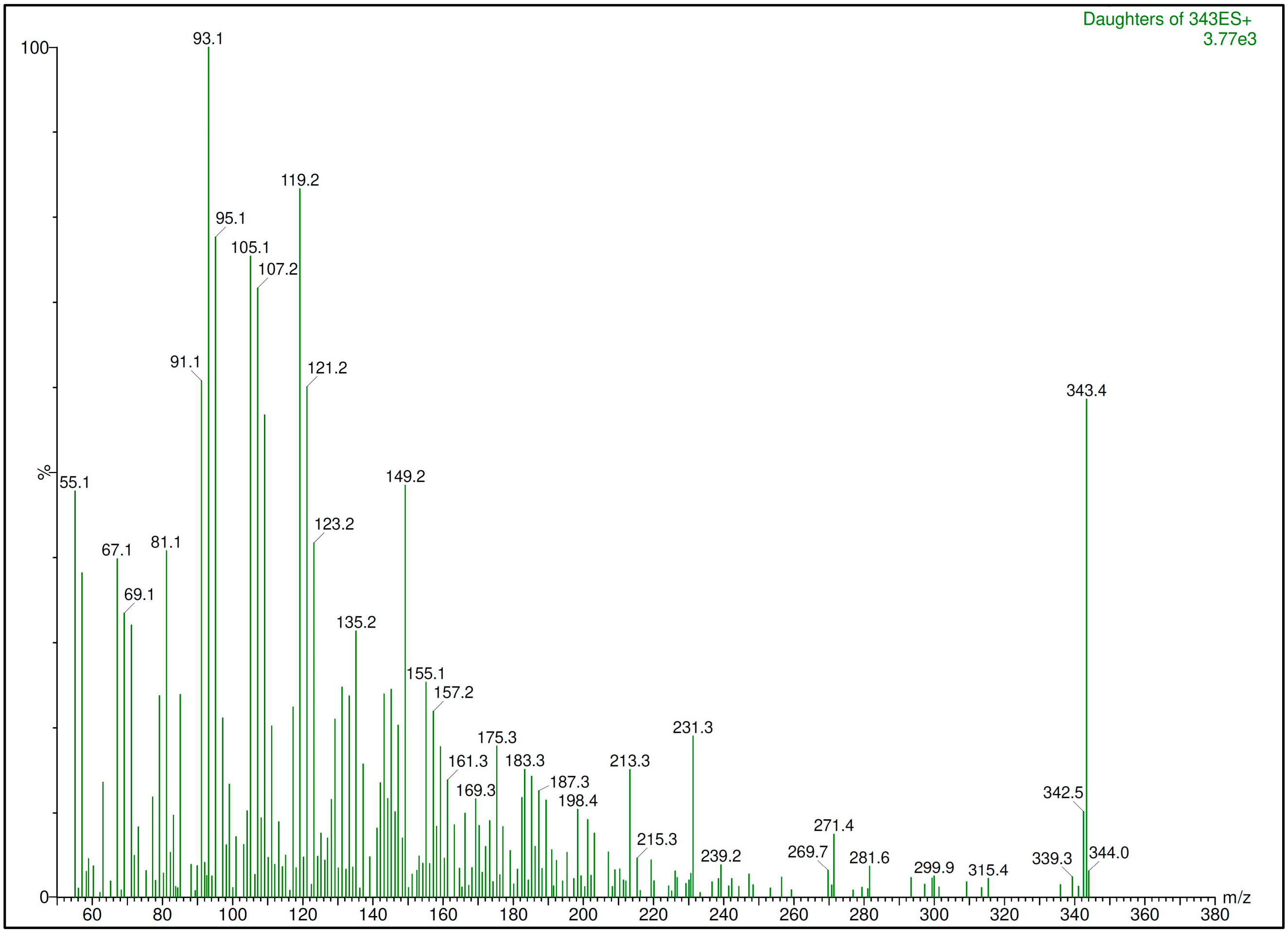

Supplement: Supplementary file 1 [file cimb-47-00053-s001.zip › cimb-3398763-supplementary.pdf]
